# Enhanced Protein Immobilization on Polymers—A Plasma Surface Activation Study

**DOI:** 10.3390/polym12010104

**Published:** 2020-01-04

**Authors:** Felicia Wieland, Richard Bruch, Michael Bergmann, Stefan Partel, Gerald A. Urban, Can Dincer

**Affiliations:** 1 Laboratory for Sensors, Department of Microsystems Engineering, University of Freiburg, 79110 Freiburg, Germany; felicia.wieland@googlemail.com (F.W.); bruch@imtek.de (R.B.); bergmann@imtek.de (M.B.); urban@imtek.de (G.A.U.); 2 Freiburg Center for Interactive Materials and Bioinspired Technologies, University of Freiburg, 79110 Freiburg, Germany; 3 Research Center of Microtechnology, Vorarlberg University of Applied Sciences, Dornbirn 6850, Austria; stefan.partel@fhv.at; 4 Freiburg Materials Research Center, University of Freiburg, 79104 Freiburg, Germany

**Keywords:** surface activation, oxygen plasma, protein immobilization, biosensors

## Abstract

Over the last years, polymers have gained great attention as substrate material, because of the possibility to produce low-cost sensors in a high-throughput manner or for rapid prototyping and the wide variety of polymeric materials available with different features (like transparency, flexibility, stretchability, etc.). For almost all biosensing applications, the interaction between biomolecules (for example, antibodies, proteins or enzymes) and the employed substrate surface is highly important. In order to realize an effective biomolecule immobilization on polymers, different surface activation techniques, including chemical and physical methods, exist. Among them, plasma treatment offers an easy, fast and effective activation of the surfaces by micro/nanotexturing and generating functional groups (including carboxylic acids, amines, esters, aldehydes or hydroxyl groups). Hence, here we present a systematic and comprehensive plasma activation study of various polymeric surfaces by optimizing different parameters, including power, time, substrate temperature and gas composition. Thereby, the highest immobilization efficiency along with a homogenous biomolecule distribution is achieved with a 5-min plasma treatment under a gas composition of 50% oxygen and nitrogen, at a power of 1000 W and a substrate temperature of 80 °C. These results are also confirmed by different surface characterization methods, including SEM, XPS and contact angle measurements.

## 1. Introduction

Point-of-care testing (POCT) systems have recently become essential tools in the healthcare sector to accelerate medical treatment decisions or diagnosis as they enable on-site measurements in resource-limited settings, such as in developing countries, in doctor’s practice or directly at home [1,2]. Therefore, the development of POCT devices takes the center stage in many different research areas, including life science, clinical diagnostics, food analysis and environmental monitoring. In the near future, traditional diagnostic tests in clinical laboratory settings will be replaced more and more by near-patient tests [2,3].

POCT systems not only have to deliver a high performance (regarding sensitivity, selectivity and turnaround times), but they also should be cost-effective without the need for bulky instrumentation (for example, for sample preparation or signal readout). In order to produce low-cost on-site testing systems in high throughput, the employed materials have shifted over the last years from silicon, glass or ceramics, used mainly for micro- and nanoelectromechanical systems, to polymers [4]. In contrast to these advanced materials, mainly requiring expensive and time-consuming fabrication processes, polymers offer a facile and cost-effective mass production of sensors. Besides, there are many varieties of polymers along with different properties, like transparency, biodegradability or flexibility. Thus, they have become the material of choice for umpteen sensor applications [5,6].

Thermoplastics—thermosoftening polymers—are widely employed in the industry for mass production. Using various replication methods, like injection molding or hot embossing, they can be shaped and reformed above a specific temperature (i.e., glass transition temperature). Unlike other polymers, thermoplastics are available in different stiffnesses, are resistant to organic solvents and provide reduced biofouling. These features favor them as substrate material, especially for disposable sensors [4]. Typical thermoplastics include polymethyl methacrylate (PMMA), polyamide (PA), polypropylene (PP) and cellulose acetate (CA). Among them, PMMA is one of the most employed thermoplastics owing to its low costs and good availability. PMMA is transparent under visible light and amorphous and offers excellent optical and thermal properties with higher resistance to sunlight. The glass transition temperature of PMMA ranges from 85 to 165 °C. Yet, PMMA has no specific functional groups to couple biomolecules [6,7].

For nearly all biosensor applications, the interplay between the biomolecules used as recognition elements (for instance, antibodies, nucleic acids or enzymes) and the substrate surface is extremely important. Hence, the applied substrate material, and the surface activation and immobilization techniques have to be considered carefully. In order to achieve an efficient immobilization of biomolecules on polymers, various surface activation methods, including chemical and physical techniques, exist [8,9,10,11].

**Figure 1:**
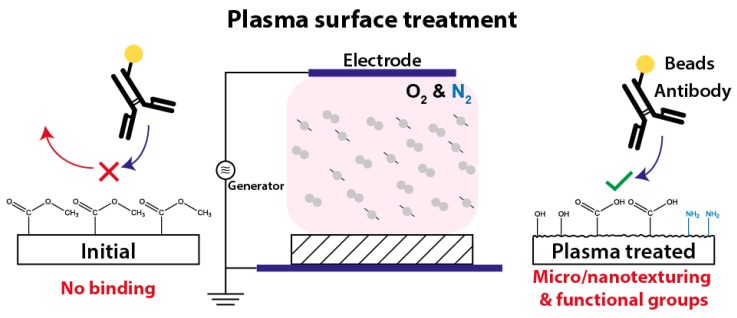
Plasma treatment of polymers results in the texturing of their surface in micro/nano range and generates functional surface groups which again facilitates the linker-free (physical and covalent) immobilization of biomolecules.

Among them, the plasma treatment of surfaces (Figure 1) is widely used for the functionalization of various materials by micro/nanotexturing and generating functional surface groups [11,12,13,14,15]. The plasma activation of a surface leads to an enhanced hydrophilicity, which is supported by the oxidation and roughness of the surface. To improve wettability, the surface energy of the liquid must be lower than of the surface itself. Polymers, in general, own a low surface energy due to the non-polar hydrogen bonds on their surface. When treated with oxygen plasma, the UV proportion of radiation breaks carbon chains, oxygen radicals are formed, and thus, wettability increases. Using nitrogen instead of oxygen, or a mixture of both, as process gas, reactive groups such as amines (NH_2_) or carboxyl groups (–COOH) can be also implemented. However, plasma treated surfaces are quickly ageing in ambient air, since the polar groups on the fresh plasma activated surfaces are not long-term stable. Therefore, the functionalization of plasma treated surfaces with biomolecules should be done immediately or the surface groups has to be derivatized chemically [12,16,17,18].

## 2. Materials and Methods

### 2.1. Chemicals

Unless otherwise stated, all chemicals are purchased from Sigma-Aldrich Chemie GmbH (Munich, Germany) and used without further purification.

### 2.2. Substrate Preparation

For the plasma activation study, different polymer foils, including a 175 µm thick PMMA foil (PLEXIGLAS^®^ Film 99524, Evonik Industries, Essen, Germany), a 75 µm thick CA foil, a 50 µm thick PA 6 foil and a 40 µm thick PP foil (Reichelt Chemietechnik GmbH + Co., Heidelberg, Germany) are used as substrate. After the plasma activation, a structured UV-tape is adhered onto the surface to define the immobilization areas for the biomolecules. The structuring of the UV-tape is realized by using a CNC cutting machine. For an easy alignment of the UV-tape, support structures, indicating the immobilization areas, are wax printed on the backside of the substrate.

### 2.3. Biomolecule Preparation and Incubation Procedure

In this work, antibodies labelled with two different methods, including gold nanoparticles (NPs) and latex beads, are employed for the generation of the optical signal. Both labelling approaches are carried out using mouse IgG antibodies (Sigma Aldrich), diluted in 10 mM PBS, pH 7.4. For all plasma activation tests, a gold conjugation kit with 80 nm gold nanoparticles (obtained from Abcam, Cambridge, UK) are employed for the antibody labelling. For this purpose, a concentration of 0.2 mg mL^−1^ of mouse IgG antibodies is conjugated by following the instructions of the labelling kit [19]. Only for the SEM investigation of the adsorbed antibodies, a conjugation kit with 400 nm red latex beads (purchased from Expedeon, San Diego, CA, USA) is used. Herein, an antibody concentration of 0.1 mg mL^−1^ is labelled according to the conjugation kit instructions [20].

The biomolecule immobilization is realized by pipetting 2 µL of labelled antibody solution into the specific area and incubating for 2 h (except the incubation time test). After the immobilization, the samples are washed by dipping in physiological PBS solution in order to remove unbound antibodies.

In order to examine the EDC/SNHS activation of carboxyl groups on the polymer surface, untreated and plasma treated PMMA foils are tested, regarding the performance of the antibody immobilization, and the findings are compared with the results of the linker-free (physical and covalent) immobilization tests. Herein, a 5-min plasma activation is done under a gas composition of 100% O_2_, at a power of 1000 W and at a substrate temperature of 80 °C. Subsequently, the PMMA foil is activated for 1 h with 100 mM EDC and 200 mM SNHS in activation buffer (0.1 M MES, 0.9% NaCl, pH 6.0) at room temperature in the dark. After a subsequent washing step by dipping the sample in the DI water, undiluted gold NP labelled antibodies in physiological PBS is incubated on the EDC/SNHS activated PMMA foil for two hours. In the case of the linker-free immobilization test, the PMMA foil is directly functionalized with antibodies.

### 2.4. Plasma Activation

For the low-frequency (at 40 kHz) plasma activation, the barrel etcher Tetra30PC (Diener electronic GmbH + Co. KG, Ebhausen, Germany) is used. The polymer foils are placed into a glass petri dish during the plasma process. First, different polymer substrates are tested, using a 3-min plasma treatment at a power of 1000 W, a substrate temperature of 80 °C under a gas composition of 100% oxygen (purity grade 5) and at a flow rate of 250 sccm. Second, the substrate temperature is ranged between 20, 40, 60, 80 and 90 °C, keeping the other plasma parameters constant. Third, the plasma power is varied between 300, 500, 700, and 1000 W, using a 3-min plasma treatment at a substrate temperature of 80 °C under a 100% oxygen environment and at a flow rate of 250 sccm. Fourth, the plasma activation time is studied, applying the optimized parameters from the previous tests along with different process durations of 1, 3, 5 and 10 min. Fifth, the gas composition is varied from 100% nitrogen (purity grade 5) to 100% oxygen by adding oxygen in different steps (20%, 50% and 80%) and at a flow rate of 250 sccm, employing the optimal plasma parameters, determined before. Finally, the efficiency of protein immobilization is studied by comparing two different immobilization methods: linker-free (adsorption along with covalent) immobilization and covalent immobilization via *N*-ethyl-*N*’-(3-(dimethylamino)propyl)carbodiimide (EDC)/*N*-hydroxysulfosuccinimide (SNHS) chemistry.

### 2.5. Surface Characterization

Optical measurements are realized by taking an image with a Samsung Galaxy A5 (2017, Samsung Electronics, Seoul, South Korea) in a custom-made Photobox (see Figure S1) and analyzing the intensity of the adsorbed bead labelled antibodies, using the software ImageJ (University of Wisconsin-Madison, Madison, WI, USA). For image analysis, the background intensity is subtracted and the calculated values are normalized. Each intensity measurement, shown in this work, is repeated eight times, unless otherwise stated.

In order to estimate the amount of adsorbed bead labelled antibodies, SEM images with a JEOL 7100F (JEOL Ltd., Tokyo, Japan) are carried out. A very low acceleration voltage of 1 keV is used to overcome charging effects and degradation of the polymer during observation. Detailed spectra of oxygen and nitrogen are recorded by XPS, using the Physical Electronics 5600ci (Perkin Elmer, Waltham, MA, USA) to study the influence of the plasma gas composition on the biomolecule immobilization. For the investigation of the surface wettability, contact angle measurements are also performed using a Drop Shape Analysis System DSA 10 MK2 (Krüss, Hamburg, Germany) along with a dispensing needle (Type #5125-1-B, acquired from Nordson EFD, Westlake, OH, USA). Here, the dynamic measurement mode is used by applying 10 µL water droplets. Each measurement is repeated four times.

## 3. Results

### 3.1. Initial Tests

**Figure 2:**
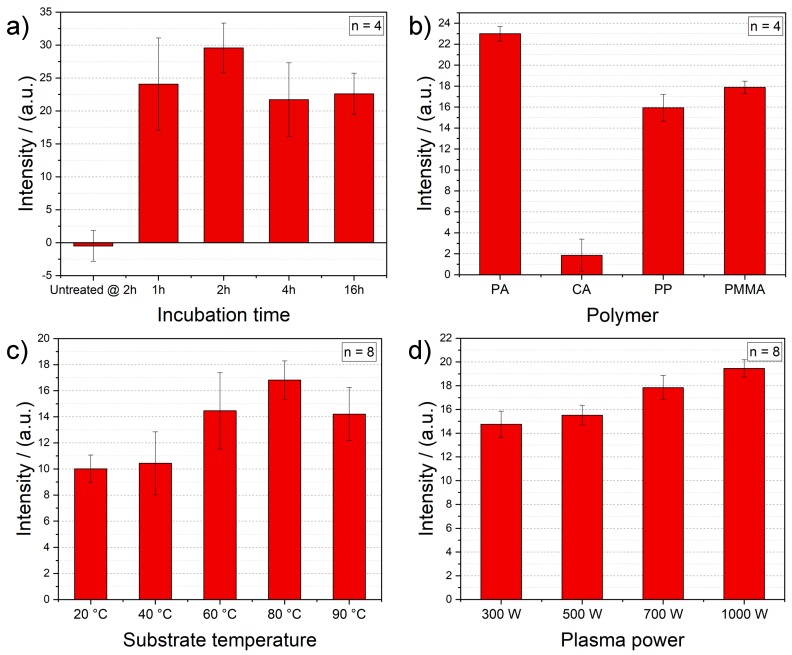
(**a**) Optimization of incubation time of the gold NP labelled antibodies on the plasma activated PMMA surface (100% O_2_, 3 min, 1000 W, 80 °C) and the background signals of 2-h incubation of antibodies on untreated PMMA foils for comparison. (**b**) Optical intensity measurement of antibody adsorption on different polymer foils after the plasma treatment (100% O_2_, 3 min, 1000 W, 80 °C). Impact of (**c**) different substrate temperatures of the plasma process (100% O_2_, 3 min, 1000 W), and (**d**) different power values of the plasma process (100% O_2_, 3 min, 80 °C) on the antibody immobilization on PMMA foils.

Prior to the plasma activation study on polymers, some preliminary tests are conducted in order to ensure the optimal conditions for the intensity measurements. First, the used antibody concentration is optimized. Herein, undiluted and different dilutions (1:10, 1:20, 1:50, 1:100 in 10 mM phosphate saline buffer—PBS) of the employed antibodies are tested after the plasma activation with initial parameters (100% O_2_, 3 min, 1000 W, 80 °C). Undiluted antibody solution offers the best option to gain a proper optical signal and thus, is chosen as standard for further experiments (result not shown). Second, the incubation time of antibodies is studied after the plasma treatment (100% O_2_, 3 min, 1000 W, 80 °C) by ranging the time between 1 and 16 h. Here, a 2-h incubation time of antibodies delivers the best outcome (Figure 2a) and thus, is selected for the final measurement protocol. The 2-h incubation results of the samples without plasma activation prove the necessity of a such surface treatment and the importance of our study.

Third, optical intensity measurements of linker-free immobilized gold nanoparticle labelled antibodies are performed on different polymer foils, such as polymethyl methacrylate, polyamide, polypropylene and cellulose acetate, after the plasma treatment (100% O_2_, 3 min, 1000 W, 80 °C). When comparing the binding performance of these foils for biomolecules (see Figure 2b), PA foils show the highest signals. PP and PMMA substrates deliver similarly good results. On the other hand, CA foils prove lower optical intensities, which indicates a relatively poor protein attachment. Even though PA and PP offers also a high immobilization capability, PMMA is used for the further plasma surface activation study due to its low costs (see Table S1).

### 3.2. Optimization of Plasma Process Parameters

After setting up the initial conditions, four different parameters of the plasma activation are examined and optimized: (i) substrate temperature, (ii) applied power, (iii) process time and (iv) the composition of the process gas. The obtained results are confirmed by various surface characterization methods, like X-ray photoelectron spectroscopy (XPS), scanning electron microscope (SEM) and contact angle measurements.

The first plasma parameter tested is the plate (i.e., substrate) temperature of the plasma reactor. Increasing the substrate temperature results in enhancing the optical intensity and thus, the immobilization of gold nanoparticle labelled antibodies (see Figure 2c). However, the results with a plasma treatment at a substrate temperature of 90 °C show lower intensities than the PMMA foils activated at 80 °C. The glass transition temperature of the employed PMMA foil is 113 °C [21], which is why this signal decrease is most probably caused by the partial damage of the PMMA foils, due to the high process temperature. Therefore, a substrate temperature of 80 °C is chosen for further plasma activation processes.

According Figure 2d, the higher the power of the plasma activation, the higher the intensity of the immobilized antibodies. This is due to the fact that by increasing the plasma power, the energy and density of electrons rise, and thus, the reactive particles have more energy. In turn, more active species result in higher reaction rates for the functionalization and modification of surfaces. Thereby, functional groups with higher binding energies are created and the etching starts sooner, both properties enhance the surface activation [22]. Therefore, the highest power value of 1000 W is selected as standard for the consecutive tests.

**Figure 3:**
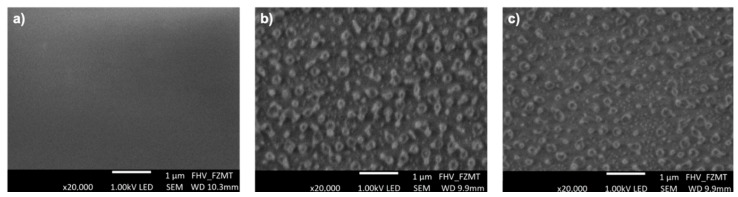
SEM images of (**a**) untreated, (**b**) and (**c**) plasma activated PMMA foils: under 100% O_2_ for 5 min, at a power of 1000 W and with a substrate temperature of 20 and 80 °C, respectively.

As mentioned, using a high-power and long plasma activation, polymers can be etched, which leads to a higher surface roughness. This again enhances the binding of biomolecules [18,23]. In order to investigate the polymeric surfaces after the plasma treatment, SEM images of native and treated (under 100% O_2_ for 5 min at a power of 1000 W and two different substrate temperatures of 20 and 80 °C) PMMA foils are undertaken and presented in Figure 3. Compared to the untreated PMMA foil (Figure 3a), the plasma treated surfaces get etched and hillock structures, in the 100 nm range, are created during the plasma process, independent of the substrate temperature (as shown in Figure 3b,c). An increase in the substrate temperature leads only to less hillocks and thus, a more efficient etching process. These hillock-like structures on the surface of PMMA are probable crystalline polymer sections that are chemically more resistant than the amorphous ones [22].

**Figure 4:**
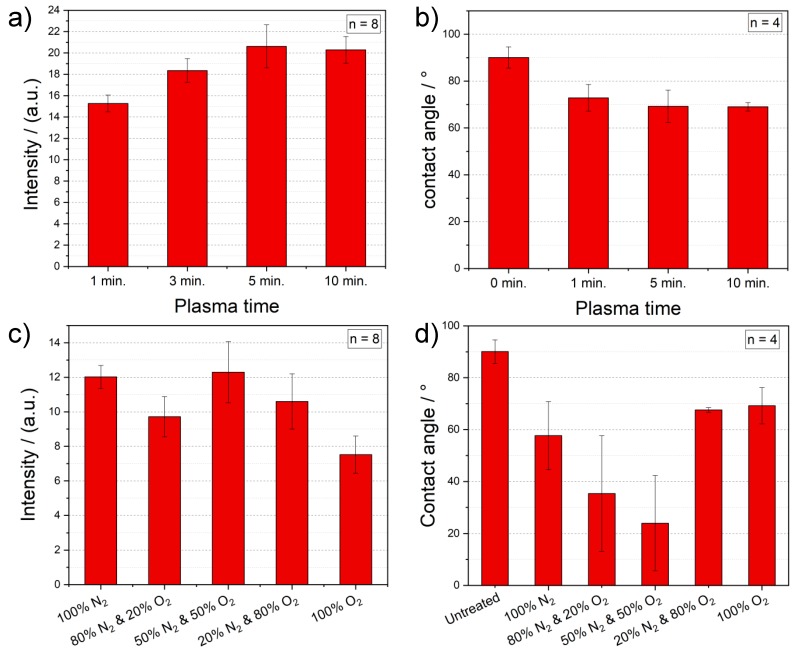
(**a**) Measured intensities of immobilized gold NP labelled antibody on plasma treated PMMA foils with different process times (100% O_2_, 1000 W, 80 °C). (**b**) Contact angle measurements of plasma activated PMMA foils with different time parameters (100% O_2_, 1000 W, 80 °C). (**c**) Optical intensity measurement of the antibody immobilization on plasma treated PMMA foils with different gas parameters for O_2_ and N_2_ (3 min, 1000 W, 80 °C). (**d**) Contact angle measurements of plasma activated PMMA foils with different gas parameters for O_2_ and N_2_ (5 min, 1000 W, 80 °C).

After the optimization of the power used in the plasma process, different plasma process times, as depicted in Figure 4a, are studied. With increasing the process duration, the ability of PMMA foils to bind antibodies increases. This is most probably caused by the enhanced surface roughness of the PMMA foil through a longer plasma activation. Yet, when reaching a process time of 10 min, the PMMA foil starts already degrading. In order to prevent any damage of the PMMA foil and have a short but effective surface activation, a 5-min plasma treatment is chosen as standard. Subsequently, contact angle measurements are done to study the surface effects, caused by different plasma activation times. The results, shown in Figure 4b, confirm the outcome of the intensity measurements, by proving a lower contact angle with increasing the plasma time. This means, that the wettability is improved by a longer plasma duration.

Finally, the impact of the gas composition during the plasma activation on the protein immobilization is analyzed. The process gas is responsible for implementing functional groups during the plasma activation. In order to implement amine groups (NH_2_) onto the polymer surface for the further improvement of the biomolecule immobilization, we systematically add nitrogen to the plasma atmosphere by decreasing the oxygen content. According to the results summarized in Figure 4c, the optical intensity increases by adding more nitrogen, until the mixture reaches 50%. Having a majority of nitrogen in the plasma atmosphere brings no improvement, but rather a deterioration. Surprisingly, PMMA surfaces activated under 100% N_2_ also prove higher optical signals and thus, a proper antibody immobilization. Nevertheless, 50% of O_2_ mixed with 50% of N_2_ delivers still the best outcome for plasma treatment of PMMA foils. These results are also underlined by the contact angle measurements, illustrated in Figure 4d. Herein, the samples plasma activated under a gas mixture of 50% O_2_ and 50% N_2_ show the lowest contact angles of around 24°.

For further analysis of the influence of the plasma gas composition, XPS measurements are performed with untreated and plasma treated (5 min, 1000 W, 80 °C) PMMA foils under 100% O_2_, 50% N_2_/O_2_ and 100% N_2_. XPS can identify all elements on the outermost layer (up to a depth of 10 nm) of the sample surface [24,25]. The plasma treatment can change the substrate chemically and physically on the outermost layer of its surface (nm-range) and in depth (μm-range) [18]. The surface effects are mainly chemical and initiated by the plasma chemistry. The in-depth changes lead to build radicals and crosslinking [12,18]. This is the case especially for thermoplastics like PMMA, which disintegrates under specific process conditions [26].

If reactive gases are present during the plasma activation, chemical bonds and compounds of the polymer as well as plasma particles can be broken and react further. For example, oxygen plasma consists of atomic oxygen, which reacts with hydrogen and carbon of the PMMA, producing reaction products like CO_2_ and H_2_O. Undergoing this reaction, functional groups like hydroxyl (OH), carbonyl (C=O) or carboxyl (COOH) can be formed on the surface [11,13]. Nitrogen plasma process is similar to the oxygen one, and can be used to implement amino (NH_2_) groups onto the polymer surface [11]. However, nitro (NO_2_), nitroso (NO) and nitrate (NO_3_^−^) groups can also be thereby formed.

**Figure 5:**
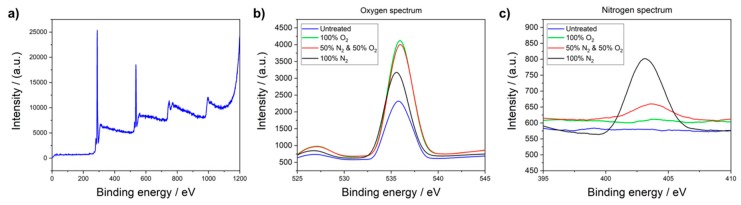
(**a**) XPS spectrum of an untreated PMMA foil. Detailed spectrum of (**b**) oxygen and (**c**) nitrogen content of plasma treated PMMA foils with different gas compositions (5 min, 1000 W, 80 °C). For the maximum oxygen content, a plasma atmosphere of 50% oxygen and 50% nitrogen is sufficient. Unlike the oxygen spectrum, in the nitrogen spectrum the highest peak is achieved by using 100% nitrogen for the plasma process.

In Figure 5a, the XPS spectrum of an untreated PMMA foil is shown, which indicates that there are strong carbon and oxygen peaks originating from the material composition itself. In addition, detailed spectra of XPS measurements of N_2_ and O_2_ are performed in order to study the effect of the plasma gas composition on the surface properties. The detailed oxygen spectrum, presented in Figure 5b, proves that oxygen is present on every PMMA foil, independent of the employed process gases. The oxygen peak of the sample, treated under 100% O_2_, is nearly the same as the one activated under 50% O_2_. The reason for the increase in the oxygen content on the PMMA foil, plasma treated under 100% nitrogen, could be that the activated species on the substrate surface react with environmental oxygen as soon as the reaction chamber is opened. Comparing the results of the detailed nitrogen spectrum, depicted in Figure 5c, there is only nitrogen present when the sample is treated with 50% nitrogen or more. The XPS results confirm both, the optical intensity and contact angle measurements. Comparing the binding energy of amine functionality with those of nitro, nitroso and nitrate groups (see Figure 5a,b), it can be clearly seen that the plasma activated polymer surface proves an amine functionality.

### 3.3. Comparison of Different Immobilization Techniques

The efficiency of the biomolecule immobilization is a crucial issue for the performance of biosensors. Thereby, the quality of the chosen immobilization strategy has a great impact [23,27]. The easiest and shortest way to couple biomolecules on a surface is the physical immobilization (i.e., adsorption). It does not require the application of any toxic and complicated linker chemistry. However, its major drawbacks are: (i) steric hindrance of biomolecules, in the case of a high-dense surface coverage, (ii) instable and random non-covalent binding, and (iii) mainly high non-specific interactions compared to other immobilization strategies [23,27].

On the other hand, the covalent immobilization techniques target at different functional groups of biomolecules such as amine groups (–NH_2_), which are suitable for a non-reversible binding of proteins on various surfaces, using a large variety of conjugation chemistry. Depending on the targeted surface groups, shortcomings of the covalent immobilization may include (i) the denaturation of biomolecules, (ii) the use of a toxic and complex linkage chemistry, and (iii) the need for longer activation and thus, incubation times [23,27].

Carbodiimide chemistry is the most commonly used technique for the covalent immobilization of biomolecules, comprising amine groups, to surfaces, containing carboxylic acids (–COOH), through a zero-length amide bond [10,28]. Here, the water-soluble EDC catalyzes the formation of an O-acylurea intermediate by activating the carboxylic acids on the surface. Yet, the O-acylurea intermediate is not stable and can either react further to an N-acylurea, a non-reactive side product, or hydrolyze back to carboxylic acid. To provide a higher stability, SNHS is often used in order to form amine-reactive NHS esters from the instable O-acylurea intermediates. In sum, two-step EDC/SNHS activation chemistry highly facilitates the immobilization efficiency of biomolecules at the physiological pH [10,29].

**Figure 6:**
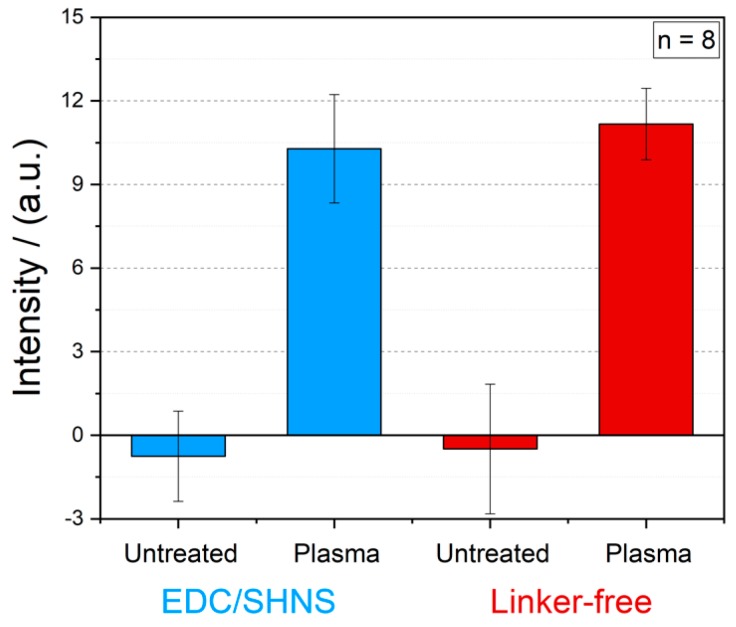
Comparison of two different immobilization techniques on the antibody immobilization: (i) EDC/SHNS based and (ii) linker-free immobilization of gold nanoparticle labelled antibodies on the untreated and plasma treated PMMA foils (100% O_2_, 5 min, 1000 W, 80 °C).

**Figure 7:**
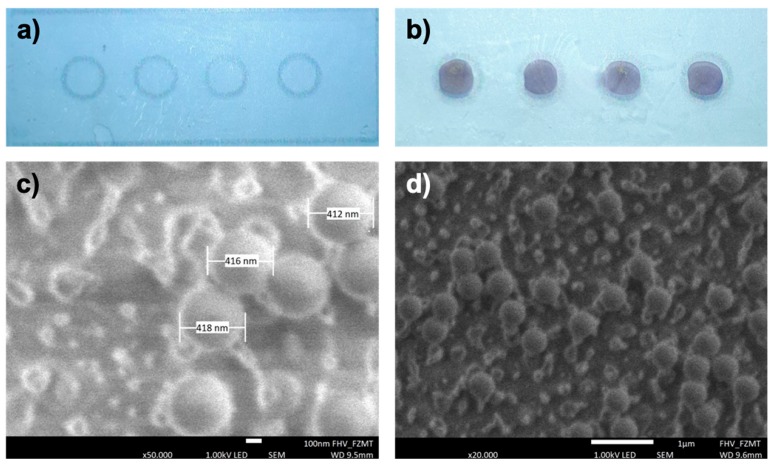
Photo of an (**a**) untreated and (**b**) a plasma activated PMMA foil after the linker-free immobilization of 400 nm bead labelled antibodies. SEM images of a single immobilization area with two different magnifications: (**c**) 50,000× and (**d**) 20,000×. The latex beads can be clearly seen in both SEM pictures and their measured diameter is around 414 nm. For the antibody density estimation, the latex beads in (**d**) are counted and extrapolated to the whole immobilization area.

In Figure 6, the results of intensity measurements of gold NP labelled antibodies immobilized by means of carboxyl-to-amine crosslinking and adsorption on PMMA foils with and without plasma activation are illustrated. It becomes clear, that the EDC/SHNS based covalent immobilization of biomolecules does not enhance the measured intensity signals and thus, a simple linker-free immobilization of biomolecules on polymers will be more than sufficient for the functionalization of the substrate. The both optical signals for linker-free and EDC/SHNS based immobilization of antibodies on untreated PMMA foils (as blank) were around zero. In order to visualize these results more clearly, images of untreated and treated PMMA foils with optimized plasma parameters after the linker-free immobilization of latex bead labelled antibodies are presented in Figure 7a,b.

### 3.4. Estimation of the Immobilized Antibody Density

For an efficient biomolecule immobilization, the number (i.e., the amount) of bound proteins is a measure. To estimate how many antibodies are immobilized in a single immobilization area (2 mm circles) on the PMMA substrate, SEM images (see Figure 7a,b) are taken. The employed latex beads labelled to the antibodies have a diameter of approximately 400 nm. The SEM picture, illustrated in Figure 7c, depicts single latex beads confirming their diameter size of around 414 nm.

The beads observed in Figure 7d belong to a part of one circle on the polymer substrate and are counted within the displayed area. Furthermore, the other three circles on the same PMMA foil are also imaged and the beads are counted as well. The average number of beads shown in the SEM images is around 35 beads per displayed area (n = 4). The SEM photograph in Figure 7d shows a circle area of 6 × 4 µm^2^ with immobilized antibodies. The complete circle has a total area of 3.14 mm^2^. Considering the number of the beads counted and assuming each bead is coupled to a single antibody, there should be more than 4.5 million bead labelled antibodies immobilized in a circle, resulting in an antibody density of 1.45 million (around 58 ng) antibodies per mm^2^. This is fully in accordance with the initial amount of incubated antibodies about 63.7 ng per mm^2^ (2 µL of 0.1 mg mL^−1^ antibody solution).

## 4. Discussion

In summary, we have demonstrated a methodical and extensive plasma activation study of different polymers in terms of the biomolecule immobilization. In this regard, the effect of various plasma parameters, such as power, time, substrate temperature and gas composition, on the immobilization efficiency has been investigated and optimized. As expected, increasing the power and the time of the plasma process result in higher optical intensities and thus, a more homogeneous and effective protein immobilization. Yet, the duration of the plasma treatment shows only a limited impact since polymers start to degrade during a longer plasma process (more than 10 min). Surprisingly, the substrate temperature plays a crucial role in the immobilization performance of the plasma activated surfaces. Herein, the best results are achieved at a substrate temperature of 80 °C (near to the glass transition temperature). Plasma treatment generates hillock-like structures on the polymer surface. An increase in the substrate temperature leads only to less hillocks and hence, a more efficient etching process. Furthermore, a mixture of 50% oxygen and 50% nitrogen as plasma atmosphere proves to have the lowest contact angle, a homogenous protein binding and thus, the highest optical signals.

Among all tested polymers, PA, PMMA and PP delivered equally good results after plasma activation. However, PMMA is chosen for the further tests, owing to its low costs and great availability. The highest immobilization efficiency on PMMA substrates is obtained with a 5-min plasma treatment at a power of 1000 W and a substrate temperature of 80 °C and under a gas composition of 50% oxygen and 50% nitrogen. Besides, two different immobilization methods, linker-free and EDC/SHNS based immobilization, on the plasma treated polymeric surfaces are compared and no significant difference in the measured optical signals are observed. According this, a simple and linker-free immobilization of proteins along with the plasma activation is sufficient for an effective and homogenous biomolecule coating on polymers.

## Supplementary Materials

The following are available online at https://www.mdpi.com/2073-4360/12/1/104/s1, Figure S1: CAD drawing of the cross-section of the 3D printed Photobox, Figure S2: Image of the whole system with integrated electronics and smartphone, Figure S3: SEM images of CA, PP and PA foils.
