# Supplementary material for: Enhanced Protein Immobilization on Polymers—A Plasma Surface Activation Study
Source: Polymers (Basel). 2020 Jan 4;12(1):104. doi: 10.3390/polym12010104 (PMC7023393; doi:10.3390/polym12010104)
Supplement: Supplementary file 1 [file polymers-12-00104-s001.zip › polymers-659559-supplementary.docx]

**Supplementary Materials: Enhanced Protein Immobilization on Polymers—A Plasma Surface Activation Study**

**Felicia Wieland, Richard Bruch, Michael Bergmann, Stefan Partel, Gerald A. Urban and Can Dincer**

**PhotoBox**

The Photobox is designed with SolidWorks 2014 (Dassault Systemes SolidWorks Corp., France) and printed out of black acrylonitrile butadiene styrene (ABS) with the 3D printer Ultimaker 3 Extended (Ultimaker B.V., Netherlands). It is printed in black in order to achieve a better absorption of the environment light.

The printed circuit board (PCB) is designed with EAGLE 8.5.0 (CadSoft Computer Ltd., Germany) and manufactured by the company Beta LAYOUT Ltd. (Germany). The soldering of the electronic components is done internally. To improve the image quality, a white LED is integrated onto the PCB in order to take pictures without the need of the smartphone’s flashlight, but only with this LED turned on.


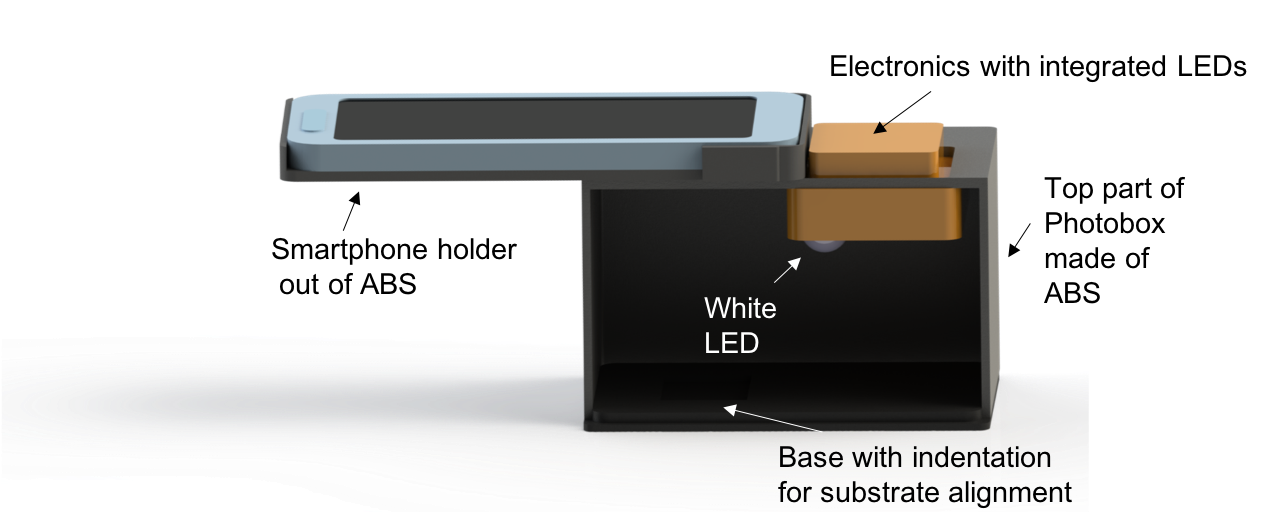


**Figure S1.** CAD drawing of the cross-section of the 3D printed Photobox with movable base for chip loading, fixed smartphone holder and integrated electronics.


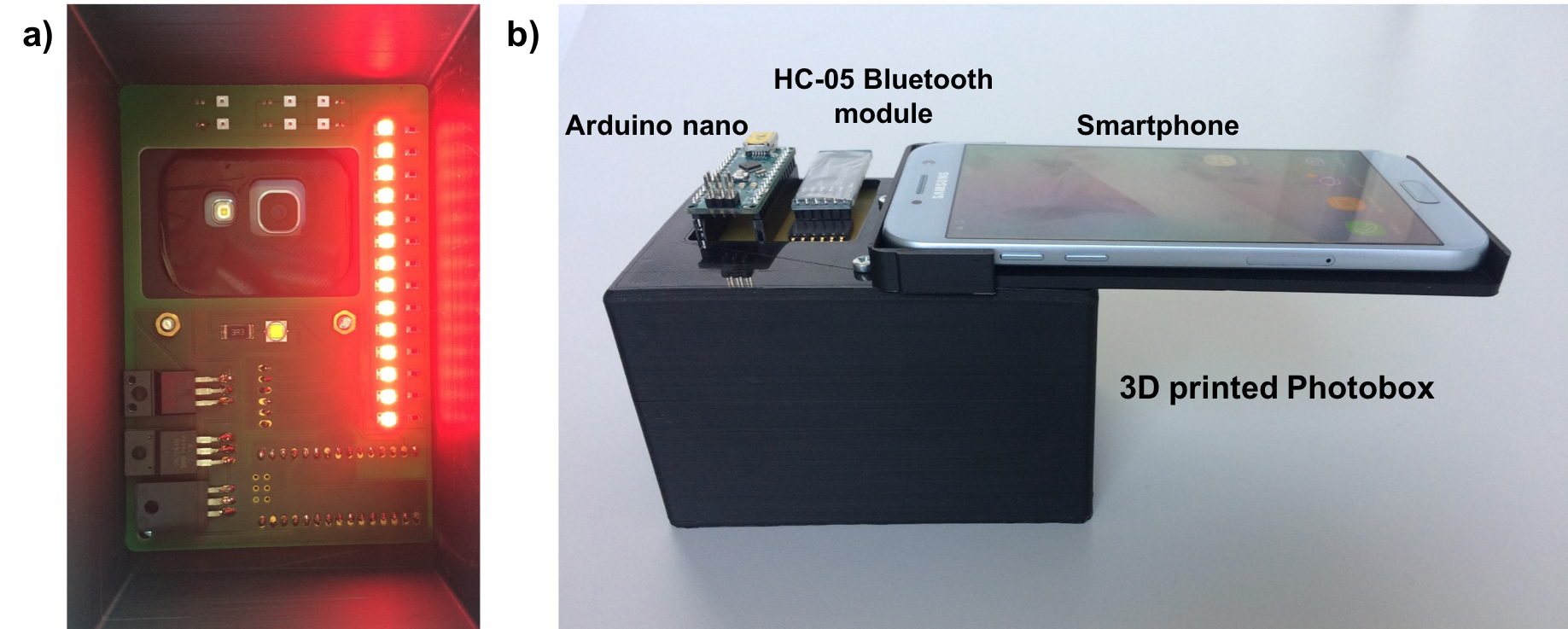


**Figure S2.** Images of a) the PCB including an Arduino nano (Arduino AG, Italy), HC-05 Bluetooth module (Aukru High-Tech Co., Ltd, UK) and different LEDs, and b) the whole system with integrated electronics and smartphone.

**SEM images of other polymers used in this work**

To study the effect of plasma treatment on surfaces of the other polymers used in this work, SEM images of untreated and plasma activated (under 100% O_2_ for 5 minutes at a power of 1000 W and two different substrate temperatures of 20 and 80 °C) PA, PP and CA foils are taken and illustrated in **Figure S3**.

According these, CA foils show a different behavior (almost no hillocks) than PMMA and PP samples after plasma activation. On the SEM images of PMMA and PP foils, the production of “hillocks” can be clearly observed. In general, an increase in the substrate temperature results only in less hillocks and thus, a more efficient etching. In contrast, PA proves a completely different surface morphology, out of fibers within 100 nm range, which enables some adsorption of biomolecules, even without plasma activation.


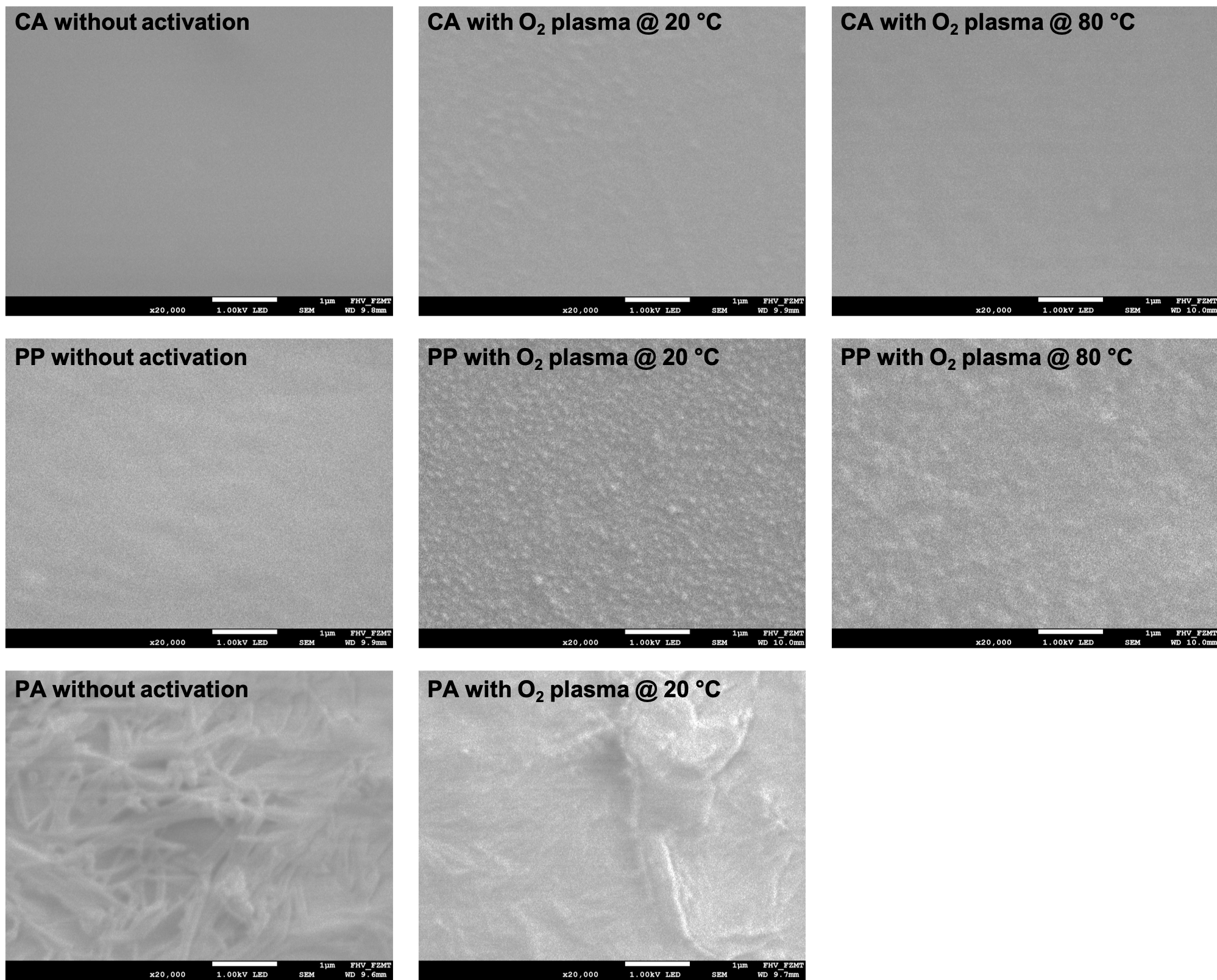


**Figure S3.** SEM images of CA, PP and PA foils: **left)** untreated, **middle)** and **right)** plasma activated foils under 100% O_2_ for 5 minutes, at a power of 1000 W and with a substrate temperature of 20 and 80 °C, respectively.

**Material Costs**

In this work, different polymer foils are used to study the effect of the plasma activation on biomolecule immobilization. The material costs are summarized in **Table S1**.

**Table S1.** Material costs of polymer foils used in this work.

|  | Cost | Size |
| --- | --- | --- |
| *PMMA* | 4.81 € | 420 $\times$ 297 cm^2^ |
| *CA* | 100 € | 300 $\times$ 300 cm^2^ |
| *PA* | 195 € | 300 $\times$ 300 cm^2^ |
| *PP* | 82 € | 300 $\times$ 300 cm^2^ |
